# Comparison of HIV-1 drug resistance profiles generated from novel software applications for routine patient care

**DOI:** 10.7448/IAS.17.4.19751

**Published:** 2014-11-02

**Authors:** Dimitri Gonzalez, Benjamin Digmann, Matthieu Barralon, Ronan Boulme, Chalom Sayada, Joseph Yao

**Affiliations:** 1 R&D, ABL TherapyEdge Spain SL, Barcelona, Spain; 2 Mayo Clinic, Virology, Rochester, USA; 3 ABL SA, R&D, Luxembourg, Luxembourg

## Abstract

**Introduction:**

Clinical laboratories performing routine HIV-1 genotyping antiviral drug resistance (DR) testing need reliable and up-to-date information systems to provide accurate and timely test results to optimize antiretroviral treatment in HIV-1-infected patients.

**Materials and Methods:**

Three software applications were used to compare DR profiles generated from the analysis of HIV-1 protease (PR) and reverse transcriptase (RT) gene sequences obtained by Sanger sequencing assay in 100 selected clinical plasma samples from March 2013 through May 2014. Interpretative results obtained from the Trugene HIV-1 Genotyping assay (TG; Guidelines v17.0) were compared with a newly FDA-registered data processing module (DPM v1.0) and the research-use-only ViroScore-HIV (VS) software, both of which use the latest versions of Stanford HIVdb (SD v7.0) and geno2pheno (G2P v3.3) interpretive algorithms (IA). Differences among the DR interpretive algorithms were compared according to drug class (NRTI, NNRTI, PI) and each drug. HIV-1 tropism and integrase inhibitor resistance were not evaluated (not available in TG).

**Results:**

Overall, only 17 of the 100 TG sequences obtained yielded equivalent DR profiles among all 3 software applications for every IA and for all drug classes. DPM and VS generated equivalent results with >99.9% agreement. Excluding AZT, DDI, D4T and rilpivirine (not available in G2P), ranges of agreement in DR profiles among the three IA (using the DPM) are shown in Table 1.

**Conclusions:**

Substantial discrepancies (<75% agreement) exist among the three interpretive algorithms for ETR, while G2P differed from TG and SD for resistance to TDF and TPV/r. Use of more than one DR interpretive algorithm using well-validated software applications, such as DPM v1.0 and VS, would enable clinical laboratories to provide clinically useful and accurate DR results for patient care needs.

**Table 1:** Minimal and maximal agreement observed between each interpretive algorithm combination per drug class

Drug class	TG vs. SD	TG vs. G2P	SD vs G2P
NRTI	79% (ABC)–99% (3TC)	62% (TDF)–99% (3TC)	61% (TDF)–99% (3TC)
NNRTI	64% (ETR)–96% (NVP)	57% (ETR)–87% (EFV)	57% (ETR)–89% (EFV)
PI	78% (LPV/r)–96% (NFV)	47% (TPV/r)–94% (DRV/r, LPV/r)	41% (TPV/r)–87% (SQV/r)

